# Ultrahigh‐Field MR‐Compatible Mechanical Tactile Stimulator for Investigating Somatosensory Processing in Small‐Bodied Animals

**DOI:** 10.1002/nbm.70105

**Published:** 2025-07-24

**Authors:** Chenyu Wang, Hirohiko Imai, Masaki Fukunaga, Hiroki Yamamoto, Yinghua Yu, Kazuhiko Seki, Takashi Hanakawa, Tatsuya Umeda, Jiajia Yang

**Affiliations:** ^1^ Graduate School of Interdisciplinary Science and Engineering in Health Systems Okayama University Okayama Japan; ^2^ Innovation Research Center for Quantum Medicine Gifu University School of Medicine Gifu Japan; ^3^ Section of Brain Function Information National Institute for Physiological Sciences Okazaki Japan; ^4^ Graduate School of Human and Environmental Studies Kyoto University Kyoto Japan; ^5^ Department of Neurophysiology National Center of Neurology and Psychiatry Kodaira Tokyo Japan; ^6^ Department of Integrated Neuroanatomy and Neuroimaging Kyoto University Graduate School of Medicine Kyoto Japan

**Keywords:** primary somatosensory cortex, small‐bodied animals, tactile stimulation device, ultrahigh‐field magnetic resonance imaging

## Abstract

Common marmosets ( *Callithrix jacchus* ), small‐bodied New World primates that share similar sensory processing pathways with human beings, have gained great interests. Their small body size allows imaging of brain activity with high spatial resolution and on a whole‐brain scale using ultrahigh‐field (UHF) magnetic resonance imaging (MRI) scanners. However, the strong magnetic field and the small size of the hand and forearm pose challenges in delivering tactile stimulation during fMRI experiments. In the present study, we developed an MR‐compatible tactile dual‐point stimulator to provide high‐precision mechanical stimulation for exploring somatosensory processing in small‐bodied animals. The study population consisted of a water phantom and three male common marmosets. Cerebral blood volume (CBV) weighted fMRI data were obtained with a gradient echo (GE), echo‐planar imaging (EPI) sequence at 7T scanner. The output performance of the device was tested by a pressure sensor. The MR compatibility of the device was verified by measuring the temporal signal‐to‐noise ratio (tSNR) of a water phantom. To test the effectiveness of tactile stimulation, we conducted block designed tactile stimulation experiments on marmosets. A one‐way repeated measures ANOVA was conducted for comparing the tSNR results. We performed one‐sample *t*‐tests to investigate the negative response of the forearm and hand stimulation with a threshold of *t* > 1.96 (*p* < 0.05). Performance tests revealed that mechanical stimulation (averaged force: 31.69 g) was applied with a delay of 12 ms. Phantom experiments confirmed that there was no significant difference in the tSNR among three (10 Hz, 1 Hz, and no‐stimulus) conditions (*F* (2, 798) = 0.71, *p* = 0.49). The CBV activity results showed that the stimulator successfully elicited hand and forearm somatosensory activations in primary somatosensory areas. These results indicated that the device is well suited for small‐bodied animal somatosensory studies.

## Graphical abstract

A dual‐point stimulator was developed to deliver precise tactile stimulation for somatosensory studies in small‐bodied animals like marmosets. Functional magnetic resonance imaging (MRI) revealed its effectiveness in eliciting hand and forearm activations in primary somatosensory areas, confirming its suitability for somatosensory studies at ultrahigh‐field MRI.

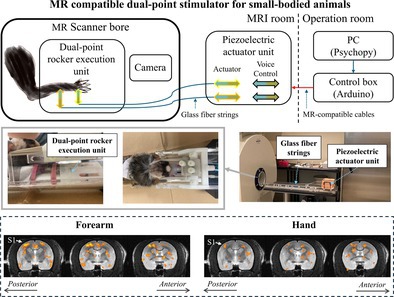

**Abbreviations**
ANOVA: analysis of variance
BOLD: blood oxygen level‐dependent
CBV: cerebral blood volume
DPREU: dual‐point rocker execution unit
DPS: dual‐point stimulator
EPI: echo‐planar imaging
FDR: false discovery rate
FOV: field of view
GE: gradient echo
GLM: general linear model
HRF: hemodynamic response function
MION: Molday ION
MR‐compatible: magnetic resonance compatible
MRI: magnetic resonance imaging
ROI: region of interest
tSNR: temporal signal‐to‐noise ratio
UHF: ultrahigh‐field
USPIO: ultrasmall superparamagnetic iron oxide

## 1 Introduction

The primary somatosensory cortex (S1) plays a complex role in processing tactile sensory information through both feedforward and feedback mechanisms [1, 2, 3, 4, 5]. Researchers often use animal models to study these functions because the sensory information processing pathways are similar to those of humans [6]. Additionally, cross‐species comparisons could provide new insights into understanding human brain functions [7]. Small‐bodied animals have great advantages in tactile sensory research because their small body sizes are suitable for imaging brain activity with high spatial resolution and on a whole‐brain scale using ultrahigh‐field (UHF) magnetic resonance imaging (MRI) scanners. The common marmoset is a small‐bodied New World primate and has gained increasing interest as an animal model for investigating primate‐specific sensorimotor neural systems [8, 9]. Marmosets have several advantages for research, including their small size, rapid maturation, and high reproductive rates [10, 11, 12, 13]. Additionally, their small lissencephalic brain simplifies the study of cortical functions and structures. The use of UHF MRI scanners has recently enabled the precise investigation of somatosensory perception in marmosets [14, 15, 16, 17]. However, the small size of their hands and forearms introduces challenges for delivering tactile stimulation in the MRI environment.

Identifying somatosensory representations in S1 is crucial for understanding how the brain processes tactile information. S1 is the initial cortical site for processing tactile information [18, 19]. In marmosets, S1 can be subdivided into areas 3a, 3b, 1, and possibly area 2 [13, 20]. Similar to humans and other nonhuman primates, S1 of marmosets contains a topographic representation of the body [21, 22, 23]. Notably, the hand has a distinct somatotopic representation in S1. Recent research has indicated that the hand area of S1 is also involved in high‐level somatosensory processing, such as spatiotemporal integration of tactile inputs [19, 24]. UHF MRI technology enables the recording of marmoset brain activity with high spatial resolution and can provide valuable insights into the neural mechanisms underlying tactile perception in S1. To fully exploit this high resolution and achieve precise mapping between brain activity and sensory input, the stimulator needs to prompt tactile‐related brain activity with precise timing and target specific body parts, such as the hand and forearm. Mechanical stimulation actuated by a motor [19, 24, 25, 26] has been shown to enable the examination of tactile sensation with high temporal and spatial precision in monkeys. However, the use of motors is restricted in the MRI scanner environment.

Currently, only electrical and pneumatic stimulation methods have been used to explore brain responses to somatosensory stimulation in marmosets via MRI experiments [27, 28, 29, 30]. Electrical pulse stimulation applied to the bilateral wrists has been used to measure blood oxygen level‐dependent (BOLD) and cerebral blood volume (CBV) hemodynamic response function (HRF) characteristics [29]. Even though electrical stimulation has high temporal precision, whether it can accurately represent real somatosensory sensations from skin deformation is debatable as electric stimulation could not generate skin deformation, which was believed as the main part to generate tactile sensation [31]. Pneumatic stimulation, which uses devices such as the Galileo tactile stimulator (Galileo Tactile Stimulator; Brainbox Ltd., Cardiff, United Kingdom), has also been employed to map the face, arm, and leg in awake marmosets [30]. However, the temporal precision of pneumatic tactile stimulation decreases as the tubing length increases. In addition, due to the small body size of marmosets and the limited space inside the MRI scanner, the puffed air may inadvertently be delivered to unintended areas. Therefore, existing stimulation devices are insufficient for accurately exploring brain activity related to somatosensory perception in marmosets.

In addition to pneumatic and electrical stimulation, piezoelectric actuators have been employed to provide tactile stimulation to explore human tactile perception in fMRI experiments. Two types of piezoelectric ceramic actuators have been used. One actuator has high‐frequency vibration [32, 33, 34]; however, its large surface area compared with the size of marmosets' hand limits its application in marmoset experiments. The other type of piezoelectric stimulation device uses a piezoelectric bimorph actuator [35]. In the previous work, we developed a tactile stimulation device based on this technology for mapping population receptive fields in the human S1 [36]. The device was developed to provide tactile stimulation to humans in a 3T scanner and has more space for the linear reciprocating motion of the carbon fiber stimulation string and a larger skin surface to fix the stimulation fiber. The actuation mechanism of this device has great potential for providing mechanical tactile stimulation to assess somatosensory processing in marmosets in combination with UHF MRI technology. However, the limited body areas of the animal restrict the use of the device since the stimulation string can be placed only along the scanner axis, and marmosets' forearm needs to also be positioned along this axis. The parallel placement of the target stimulation area and the actuator's output direction introduces a new challenge, necessitating a mechanism to convert the direction of the applied force from parallel to vertical for effective delivery of tactile stimulation.

In this work, we developed a two‐channel MR‐compatible tactile stimulation device capable of delivering stable stimuli with high temporal and spatial precision for recording brain activity in anesthetized marmosets. Building on our original device, we designed a unique double‐rocker unit to deliver mechanical stimulation to the hand and forearm of marmosets in a small‐bore UHF MRI scanner. First, we tested the output performance of the device to ensure that it did not interfere with the fMRI results. Next, we conducted fMRI experiments with marmosets to determine whether the stimulation provided by the device activated the somatosensory cortex.

## 2 Materials and Methods

### 2.1 Composition of the Dual‐Point Stimulator (DPS)

**FIGURE 1:**
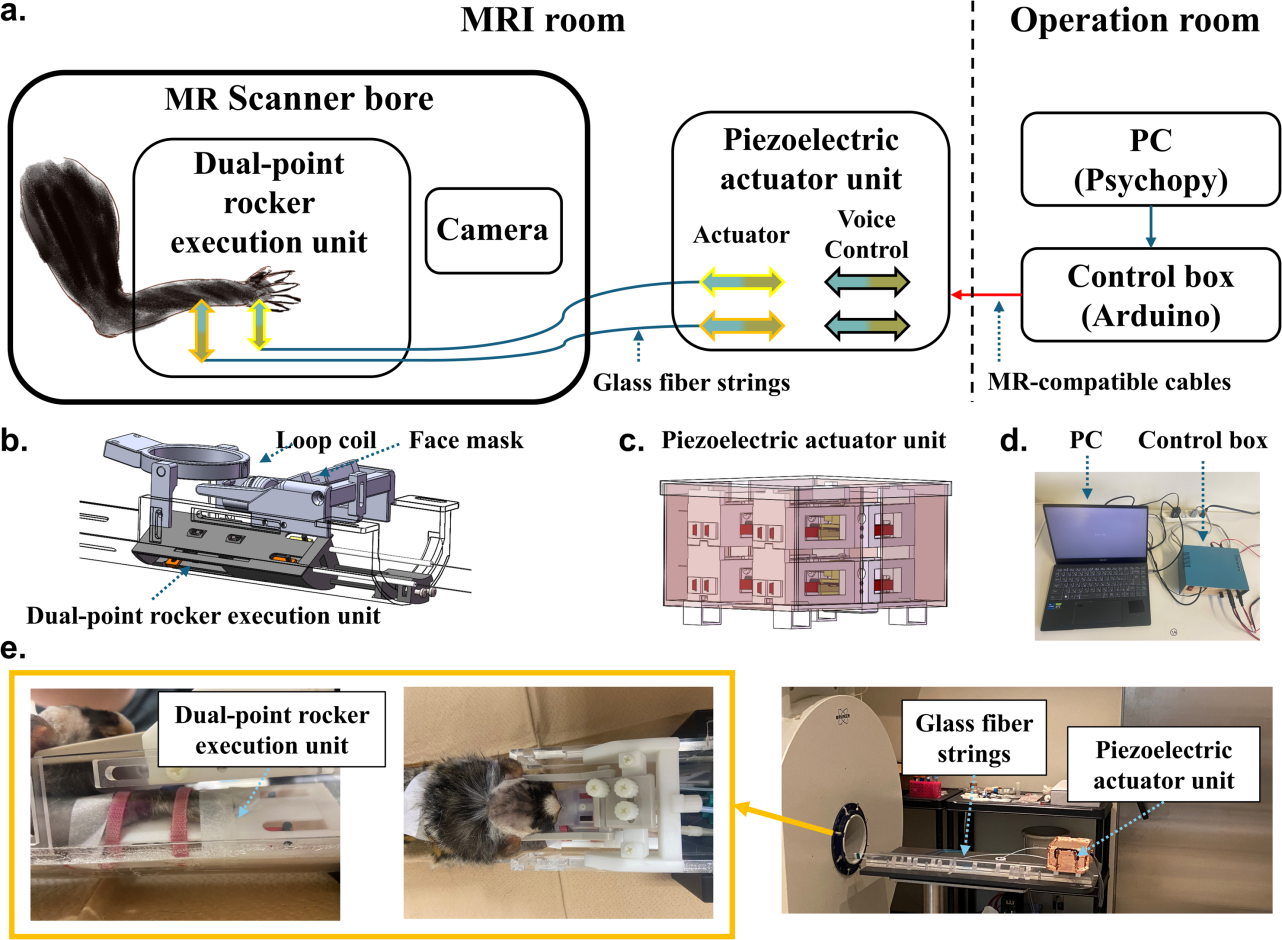
Overview of the dual‐point stimulator (DPS) and its arrangement in fMRI experiments. (a) Illustration of the overall DPS. The blue line represents the fiberglass strings that are used to transmit force. The red line expresses the MR‐compatible cables that are used to send high‐voltage control and drive signals. (b) Illustration of the arrangement of the dual‐point rocker execution unit, loop coil, and face mask in the marmoset fixation frame placed in the MR scan bore. (c) Illustration of the piezoelectric actuator unit. (d) Illustration of the control system consisting of a control box and a PC. (e) Photograph of the arrangement of the DPS. The orange frame shows the arrangement of the dual‐point rocker execution unit placed inside the MR scan bore.

The DPS was designed to provide two‐channel tactile stimulation to the hand and forearm of marmosets during the fMRI experiments. Each channel independently generated mechanical stimulation, creating a skin indentation to elicit tactile‐related brain activity in marmosets. To meet these requirements, the DPS was manufactured using MR‐compatible materials. It consisted of a dual point rocker execution unit (Figure 1b) and a piezoelectric actuator unit (Figure 1c). During the experiment, the rockers of the execution unit were pressed against the skin to generate mechanical tactile stimulation. The mechanical stimulations generated by the piezoelectric actuator unit were transmitted to the execution unit through 1.3‐m fiberglass strings. The piezoelectric actuated unit was driven and controlled by a control unit (Figure 1d) placed outside the scanner room. The control signal was transmitted via 10‐m MR compatible cables, with the shielding layer of the cable grounded. An overview of the DPS and its arrangement in the fMRI experiment is shown in Figure 1a,e.

### 2.2 Piezoelectric Actuator Unit

**FIGURE 2:**
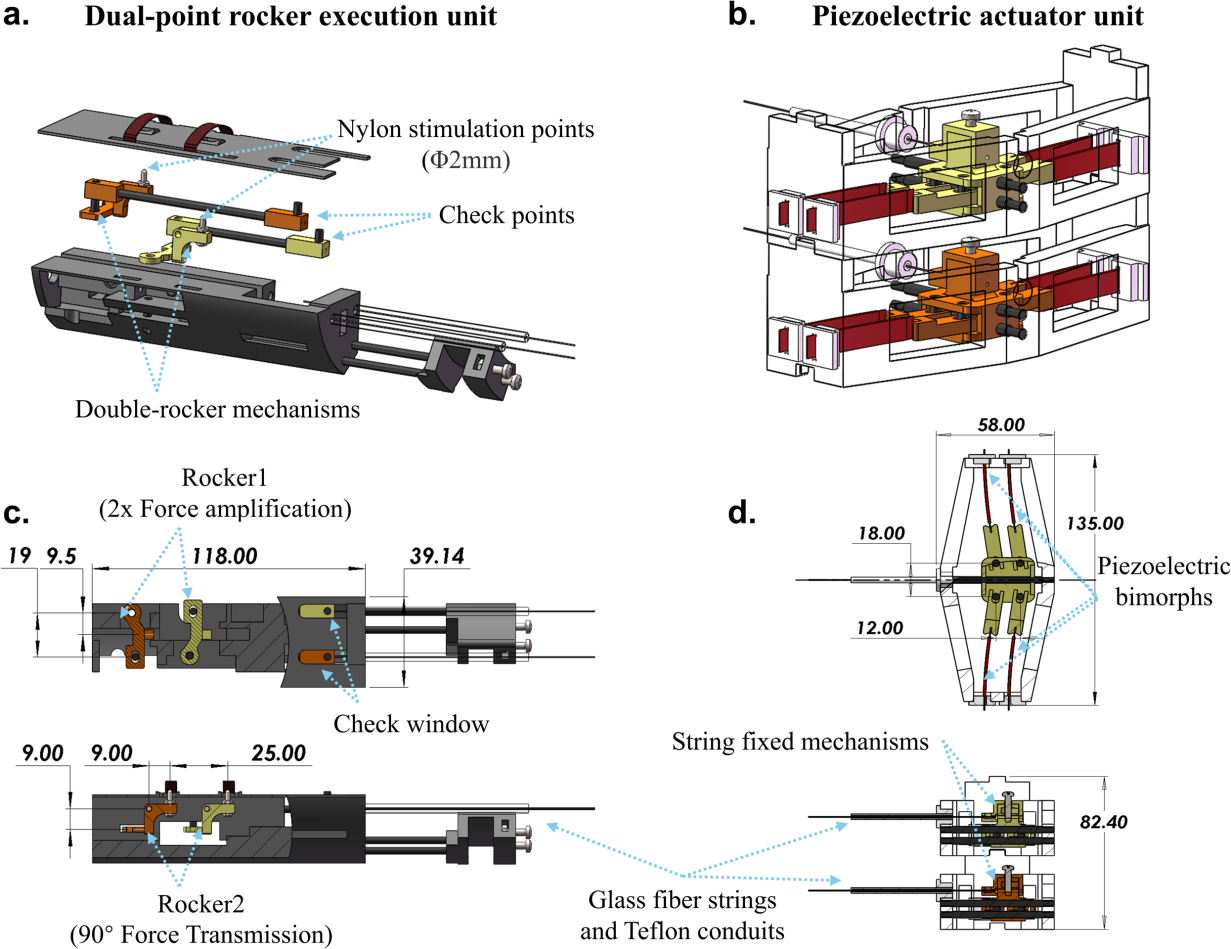
Composition of the dual‐point stimulator (DPS). (a) Composition of the dual‐point rocker execution unit. (b) Composition of the piezoelectric actuator unit. (c) Arrangement of the two rockers in the dual‐point rocker execution unit. (d) Arrangement of the 4 piezoelectric bimorphs and the string fixed mechanisms in the piezoelectric actuator unit.

For actuating two‐channel rockers of the execution unit, we developed a piezoelectric actuator unit, which is shown in Figure 2b. We chose piezo bimorphs, which are MR‐compatible actuators [35, 36], as the driving source for the system. Since the piezo bimorphs generate sound noise during operation, we built two additional identical bimorph sets to operate during the no‐stimulation periods of the experiment, providing background sound noise. The device contained four independent channels: two channels were connected to the execution unit to provide mechanical stimulation, whereas the other two channels remained disconnected from the execution unit to provide voice control functions. In the previous design [36], the horizontal‐placed paired piezoelectric bimorph actuator exhibited stable displacement (2.64 mm) over a wide range of vibration frequencies (1–30 Hz). This output performance demonstrated its potential to drive the dual‐point rocker–slider execution unit. To better realize the stimulation process, we set four piezoelectric bimorphs in one channel (Figure 2d). Instead of selecting the push part in the reciprocating motion of the actuator as the percussive stimulation, we selected the pull part as the stimulation motion in this case because the pull motion exhibited much better mechanical performance in wire transmission than the push motion. Additionally, to further decrease the impact of the noise generated by the actuators on the MRI signals, we accommodated the two sets of actuator parts in a fixation box covered with copper foil. The voice control actuator was positioned behind the device actuator inside the house of the piezoelectric actuator unit.

### 2.3 Dual‐Point Rocker Execution Unit (DPREU)

The DPREU was designed to fulfill two functions: (1) deliver and amplify mechanical stimulation to the hand and forearm of marmosets at predetermined locations and (2) stabilize marmosets' hand and forearm to ensure the stability and reproducibility of the fMRI experiment. The size and components of the DPREU are shown in Figure 2a. In accordance with the forearm and hand size of marmosets, we chose M2 nylon screws (diameter of 2 mm) as the stimulation points. The distance between the two stimulation parts was set to 25 mm to ensure that the stimulation locations were the palm and forearm. To maintain the same output performance of the two channels, the sizes of the rocker–slider mechanisms of these two channels were identical.

**FIGURE 3:**
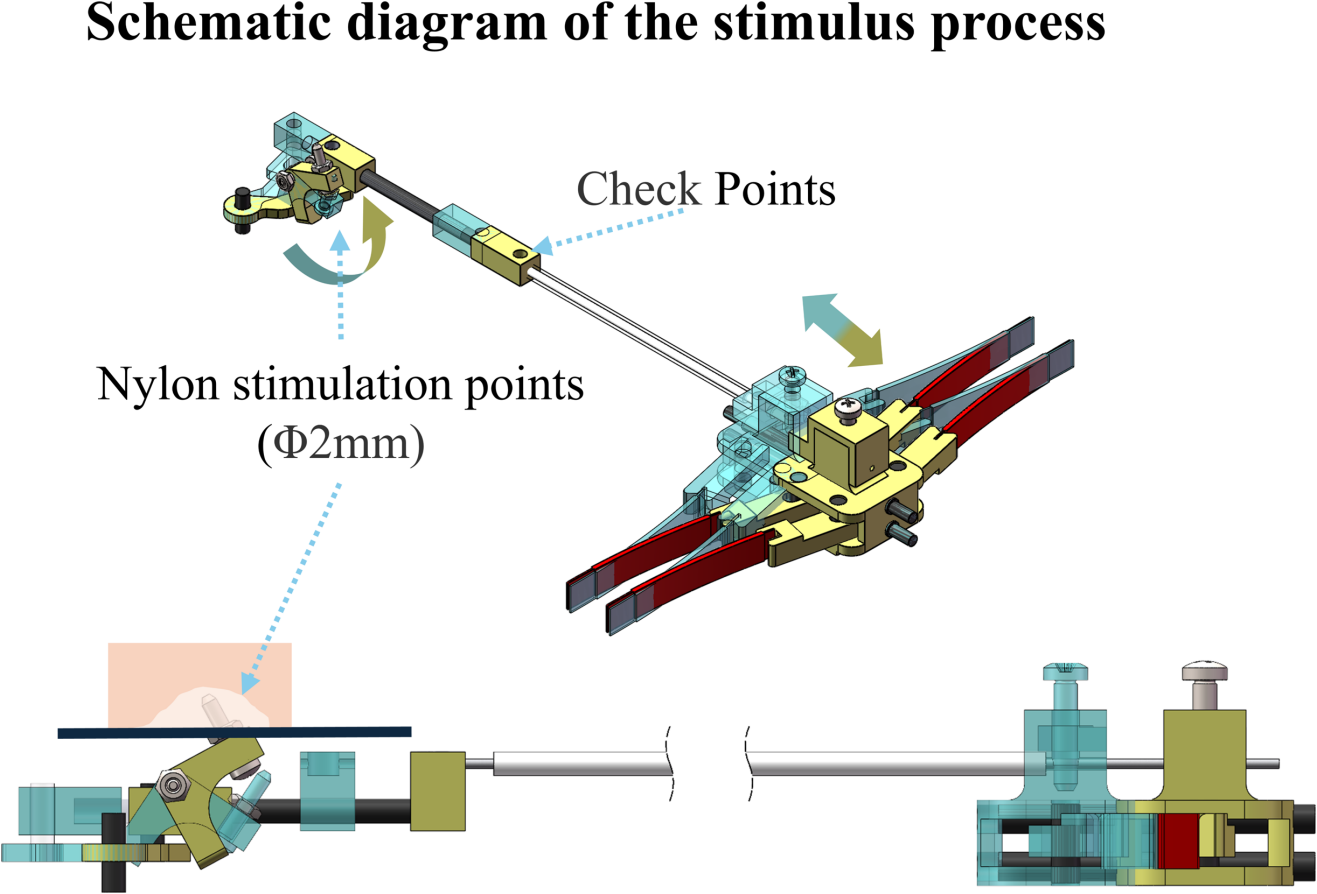
Mechanism of the stimulus process; blue represents the standby position, and yellow represents the output position.

Figure 2c shows the size of the two rockers containing the DPREU. The string pulled by the piezoelectric actuator was set to pull the rocker1 at the tail, whereas the middle part of rocker1 was set to pull rocker2. This design amplified the output force. Specifically, we designed a rocker2 with a 90° angle to convert the horizontal force into a vertical stimulation force. This design overcame the limitation of the piezo bimorph actuator, which could only generate force in a single direction. To improve motion stability, we selected a carbon fiber bar with a 3‐mm diameter and cut it to the required length to serve as the transmission axis. The motion principle of the double‐rocker mechanism is shown in Figure 3. To ensure that the stimulation process was visible during the experiment, we designed two check windows at the front of the DPREU. Two bars were connected to the transmission axes of the two channels, accordingly, and moved in sync with the fiberglass string. Using an MR‐compatible camera, we could observe the stimulation process by monitoring the performance of the bars through the check window.

The fixation function was achieved using two parts: the band part and the housing fixation part. The two bands were affixed to the housing of the DPREU to secure marmosets' hand and forearm to the stimulation locations. A curved plate with a height of 1 mm and a width of 6 mm was positioned at the hand position to ensure direct contact between the marmoset palm and the stimulation point. To ensure that marmosets remained in a relatively comfortable position during the experiment, we designed a housing fixation part to secure the DPREU at a 45° angle with the horizontal panel. To maintain the stability of the entire system, the housing fixator was clipped onto the scanner bore.

### 2.4 Control Unit

Figure 1d shows the composition of the control unit. The process of all stimulus presentations was controlled by the PsychoPy system. The system was triggered by a custom‐made mouse trigger box; this box was responsible for responding to the TTL signal from the MRI scanner. Since the four‐channel piezo bimorphs were driven by high‐voltage signals, we chose a relay module to actuate the DPREU. The on and off situations of these four channel relays on the relay module were independently controlled by 4 I/O ports. The output frequency precision of stimulation was attained by the “millis()” function on the Arduino Uno. The Arduino Uno communicated with the PC and received the channel selection signal using the serial port communication.

### 2.5 Experimental Verification

To better assess the output performance of the DPS, the output forces at l, 5, and 10 Hz were measured. To test the stimulus performance delivered to marmosets, a plate containing a thin‐film pressure sensor (ViaGasaFamido, RP‐C‐MK01X pressure sensor) was affixed to the upper surface of the DPREU housing to record the output force. Real‐time force data were sent to the PC via a serial port at a baud rate of 9600.

**FIGURE 4:**
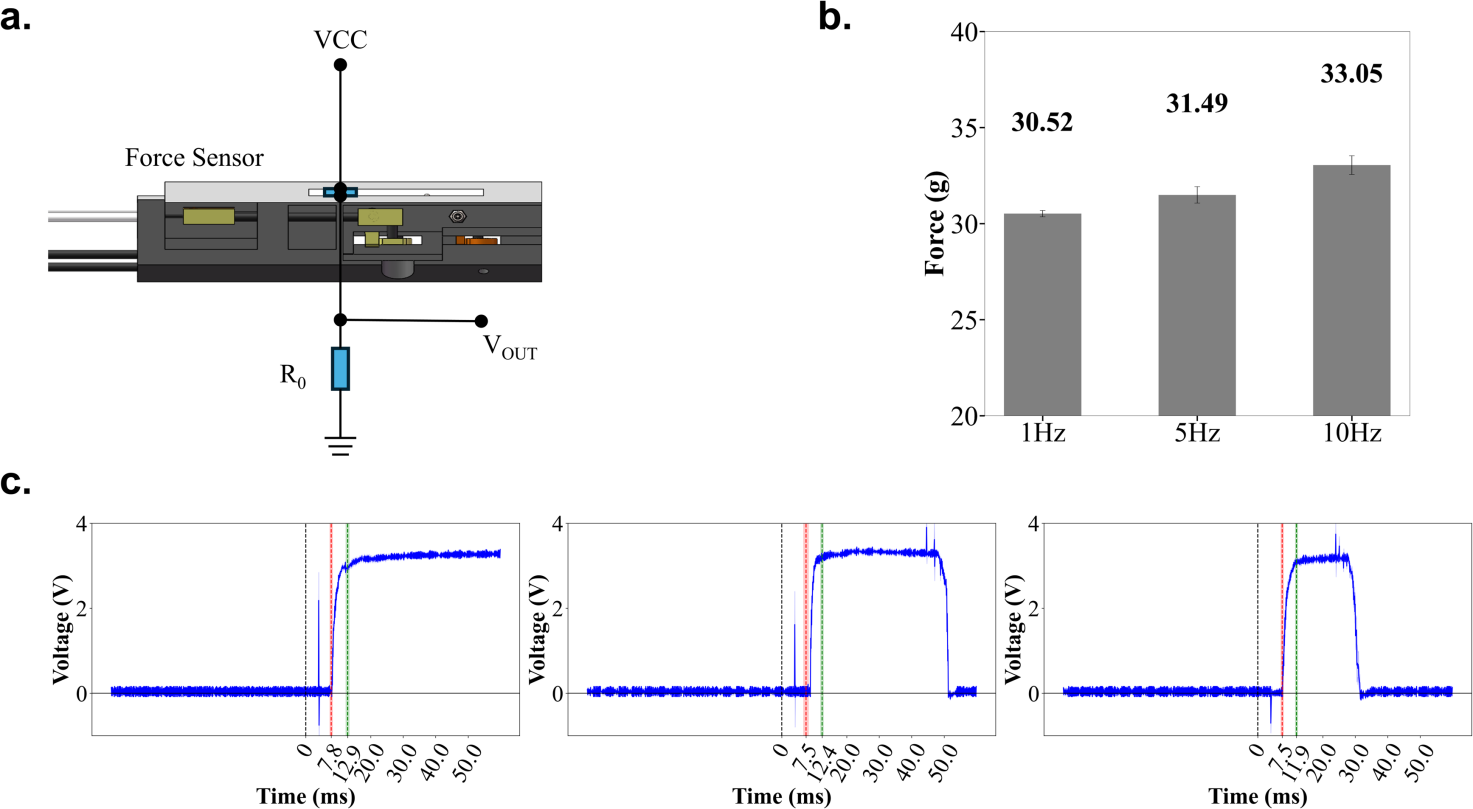
Performance of the dual‐point simulator (DPS). (a) Illustration of the arrangement of the force sensor during the test of the output force and output delay of the DPS. (b) Output forces at the three vibration frequencies. The error bars indicate the standard error of the mean (SEM). (c) Output delay results shown on the oscilloscope, and the time axis is magnified to the millisecond level; the duration between two time points represents 0.1 ms. The blue line represents the voltage of the VOUT, indicating the temporal change in force. The 0 point represents the onset time of the control signal generated by Arduino. The red line indicates the onset time of the stimulation process of the DPS. The green line represents the increase in stimulation force that reaches the stable output conditions. The widths of the red or green lines indicate the standard error of the mean (SEM).

Since the pressure sensor could not capture force changes and output delays at the millisecond level, we constructed a recording circuit using the same film pressure sensor (DF9‐16) to determine the temporal precision of the DPS. This sensor was attached to the upper surface of the housing, as shown in Figure 4a. The V_OUT_ signal from the R_0_ (100 KΩ) resistor was recorded using an oscilloscope (RIGOL DS1000Z) during the output process across three vibrational frequencies.

**FIGURE 5:**
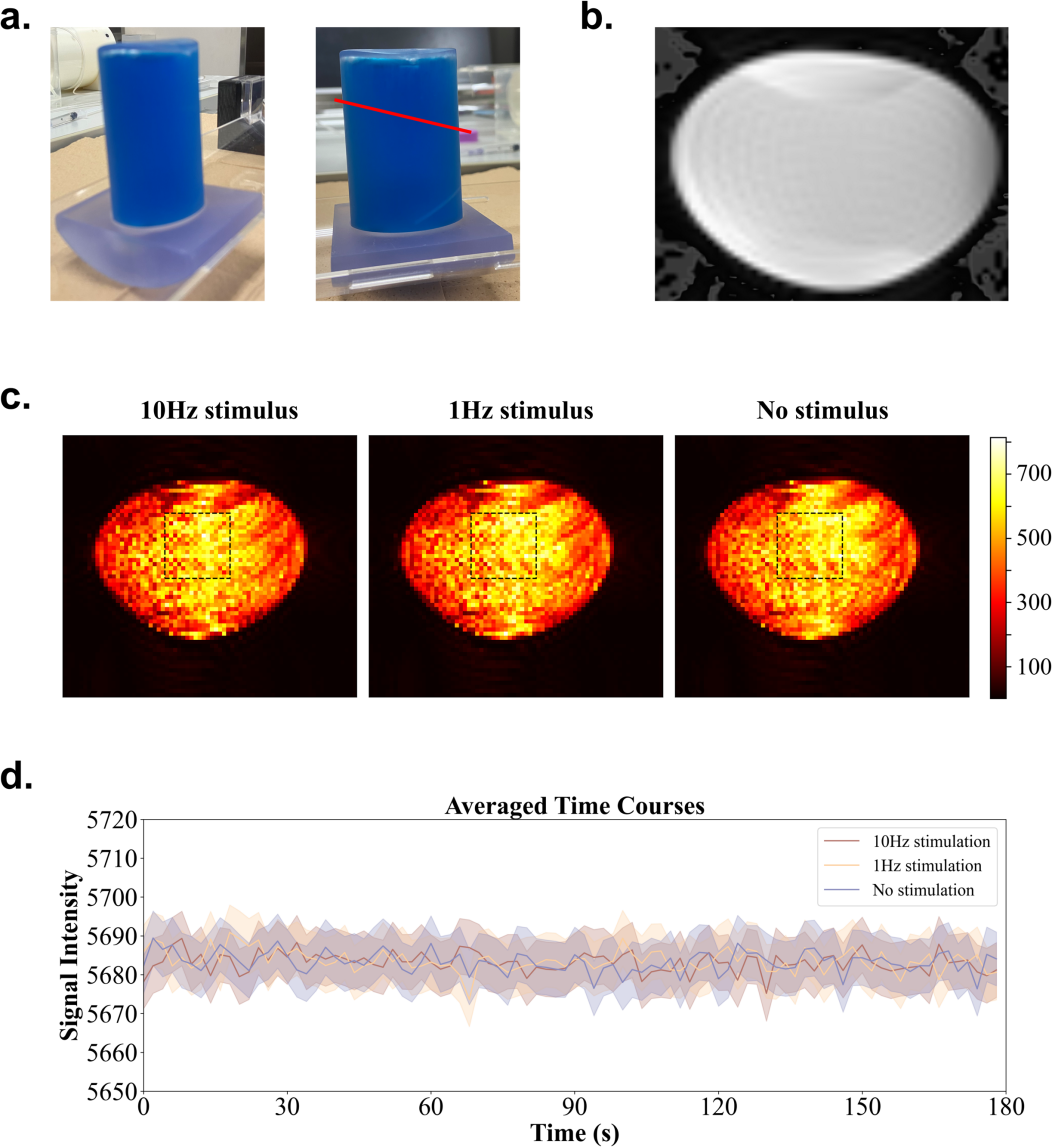
Illustration of the water phantom experiment. (a) Photo of the water phantom used in the temporal signal‐to‐noise ratio (tSNR) experiment. The red lines indicate the scan field of view (FOV). (b) Illustration of the scanned image of the water phantom. (c) tSNR results of the water phantom under 1 Hz, 10 Hz, and no stimulus conditions. The black dotted squares indicate the regions of interest (ROIs) used to calculate the tSNR. (d) Averaged time courses within the ROI for the three conditions (1 Hz, 10 Hz, and no stimulus). The shaded areas represent the standard error of the mean (SEM).

The MR compatibility of the device was verified by measuring the temporal signal‐to‐noise ratio (tSNR) of a water phantom (Figure 5a) using the same MRI scanner used in the marmoset experiment. The tSNR measurement was performed with a 7T/20‐cm bore magnet (Bruker‐Biospin, Billerica, MA, USA). A saddle coil with an inner diameter of 86 mm was used as a transmit coil, and the MR signal was acquired from one loop coil (Takashima Seisakusho Co. Ltd., Tokyo, Japan). One slice placed at the center of the phantom was scanned to calculate the tSNR. The image of the phantom, which is shown in Figure 5b, is derived from a single‐shot gradient echo‐planar imaging (GE‐EPI) sequence (FOV, 60 × 50 mm^2^; matrix, 72 × 64; slice thickness, 1 mm; voxel size, 0.833 × 0.781 × 1.0 mm^3^; acquisition bandwidth, 250 kHz; TE, 10 ms; TR, 2000 ms; flip angle, 60°). The tSNRs were separately assessed at low‐frequency (1 Hz), high‐frequency (10 Hz), and no‐stimulus conditions. These three stimulation conditions were presented in a repeated sequence of 1‐min‐long blocks: 10 Hz (1 min)—1 Hz (1 min) )—no stimulus (1 min), repeated three times for a total duration of 9 min. The tSNRs under the three conditions were extracted from a region of interest (ROI) (16 × 16 = 256 pixels) placed at the center of the phantom for statistical analysis to determine the effect of the device's operation on image quality. A one‐way repeated measures analysis of variance (ANOVA) was conducted for comparing the tSNR of the three conditions.

### 2.6 Animal Preparation

All experiments were approved by the experimental animal committee. Three adult male common marmosets ( *Callithrix jacchus* ; Marmoset #1: aged 7 years; body weight 340–360 g; Marmoset #2: aged 2 years; body weight 373–393 g; Marmoset #3: aged 4 years; body weight 372–392 g) were used. All interventions and animal care procedures were performed in accordance with the institutional guidelines for animal experiments and the National Institutes of Health Guide for the Care and Use of Laboratory Animals.

On the day of the experiment, initial sedation was induced by a small dose of ketamine (20 mg/kg) and xylazine (0.4 mg/kg), and anesthesia was maintained using isoflurane (2.5%), which was inhaled via a facemask. After the marmoset received cannulation into the leg vein, anesthesia was switched to a constant intravenous infusion of dexmedetomidine (5 μg/kg/h) and 0.5% isoflurane. A dose of 20 mg/kg of 30‐nm ultrasmall superparamagnetic iron oxide (USPIO) particles (Molday ION [MION], Biophysics Assay Laboratory Inc., USA) was injected 30 min before the start of the fMRI scan. With high‐dose MION, fMRI scans have focused mainly on the CBV reaction. Lower concentrations of isoflurane may cause the marmoset to begin waking, indicated by an increased respiratory rate and visible bodily movements. When these signs were observed, the isoflurane concentration was immediately increased to maintain sedation.

### 2.7 Stimulation and Tasks

**FIGURE 6:**
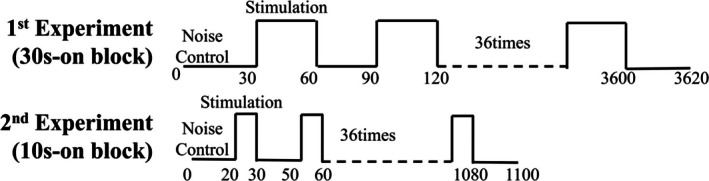
Two types of off/on block paradigms. The 30‐s‐on experiment consisted of successive 30 s off periods followed by 30 s stimulation blocks, whereas the 10s‐on experiment involved successive 20 s off periods followed by 10 s stimulation blocks. During the stimulation periods, one of three mechanical stimulations (1, 5, or 10 Hz) was delivered to the marmosets' forearm or hand. During the off periods, the voice control actuator operated at the same frequency as the subsequent stimulation to generate background sound noise. This off/on cycle was repeated 36 times in each run of each experiment, and the frequency and location order was randomized.

We stimulated the right forearm and hand with our device to investigate whether our device could elicit somatosensory‐related brain activity in the marmoset during anesthesia. Marmoset #1 joined 30‐second‐on (30s‐on) and 10‐second‐on (10s‐on) block paradigms over 2 days (Figure 6). Marmosets #2 and #3 joined a 10s‐on block paradigm 1 day. On the 30s‐on block paradigm experiment day, we conducted three fMRI runs using a 30‐second‐on block paradigm (36 min per run), and each run consisted of 36 cycles of 30 s off and 30 s on stimulation blocks targeting the hand or forearm. On the 10s‐on block paradigm experiment day, we performed six runs using a 10s‐on block paradigm (18 min per run), and each run consisted of 36 cycles of 20 s off and 10 s on stimulation blocks. During the stimulation periods of each run, one of three mechanical stimulations (1, 5, or 10 Hz) was delivered to the marmosets' right forearm or hand. The duty ratio for each frequency was 0.2; thus, the duration of each stimulation was 200 ms at 1 Hz, 40 ms at 5 Hz, and 20 ms at 10 Hz. During the noise control periods, the voice control actuator operated at the same frequency as the upcoming stimulation to provide background sound noise. Each experimental day (30s‐on experiment and 10s‐on experiment) used a distinct stimulation order, and the runs on the same day followed a consistent order.

### 2.8 MRI Data Acquisition

The fMRI experiment was conducted using the same MR scanner and the same coil for the tSNR measurement. Both the 30s‐on and 10s‐on experiments used the same EPI scan settings. T2*‐weighted fMRI data were obtained with a single‐shot GE‐EPI sequence (FOV, 25.875 × 23 mm^2^; matrix, 72 × 64; slice thickness, 1 mm; voxel size, 0.36 × 0.36 × 1.0 mm^3^; acquisition bandwidth, 250 kHz; TE, 10 ms; TR, 2000 ms; flip angle, 60°). After the EPI scans were performed each day, an additional T2 structure run was conducted to obtain a clear structural image of the marmoset with a rapid acquisition with relaxation enhancement (RARE) sequence with the following settings: TR, 5000 ms; rare factor, 8; effective TE, 39 ms; FOV, 40 mm × 40 mm; voxel size, 0.156 × 0.156 × 1.0 mm^3^; slices, 36; bandwidth, 46,875 Hz; the number of averages, 2. Marmosets #2 and #3 awoke during the anatomical scan, and we only got Marmoset #1’s T2 structure images.

### 2.9 fMRI Data Analyses

#### 2.9.1 GLM Analysis of Task fMRI Data for Activation

The EPI dataset was processed with the AFNI [37] to observe the brain activations related to forearm and hand stimulation. Then, motion correction and volume registration were applied. The time series of each voxel was scaled to a mean of 100 for future analysis. Then, a 1‐mm spatial smoothing was applied. Additionally, a high‐pass filter (0.016 Hz for the 10s‐on block and 0.008 Hz for the 30 s‐on block) was applied to remove the lowest noise frequencies. Using the AFNI “3dDeconvolve” command, a general linear model (GLM) was fitted to the preprocessed EPI data.

Initially, we focused on the brain's somatotopic mappings of the hand and forearm. The onset and duration of the two conditions (hand and forearm) were convolved with the MION response model [38, 39] of the AFNI to estimate task‐related characteristics of the time series. The 30s‐on and 10s‐on runs were analyzed with the “MION (1, 30)” model and the “MION (1, 10)” model, respectively. We performed a one‐sample *t*‐test to investigate the negative response of the forearm and hand with a threshold of *t* > 1.96 (*p* < 0.05).

#### 2.9.2 Center of Mass Analysis

To investigate the location of the hand and forearm areas, the centers of mass of each area in S1 showing significant activity in response to hand or forearm stimulation were extracted. To compare the coordinates across three marmosets, we aligned the centers of mass of Marmoset #2 and #3 to the Marmoset #1 structural space by Functional MRI of the Brain's linear registration tool (FLIRT) of FSL. Two‐tailed paired‐sample *t*‐tests were conducted to determine the positions of the centers of mass of the hand and forearm somatosensory representation areas in S1. The *xyz* coordinates of the center of mass of the significant activation regions in S1 were analyzed using *R*. The normality of the datasets was assessed using the Shapiro–Wilk test. A significant level of *p* = 0.05 was set for the paired‐sample *t*‐test comparing the positions of the centers of mass of the hand and forearm somatosensory representation areas. *P*‐values were corrected using the false discovery rate (FDR) method.

#### 2.9.3 Time Series Analysis

To investigate the brain response characteristics to various frequencies in the marmoset brain, we conducted a time series analysis. First, we defined voxels of interest (ROIs) that exhibited significant activations in S1. We then extracted the functional time series from these specified regions using the scaled EPIs at these ROIs. Next, these signals were deconvolved using a “TENTzero” function in the “3dDeconvolve” tool of AFNI to model the signal changes for each task.

## 3 Results

### 3.1 Development and Test of the DPS System

The DPS system was developed to elicit brain activation in small‐bodied animals, with the following three characteristics: (1) small and tight body size to be installed within the small bore of the scanner, (2) stable output performance within a dedicated range; and most importantly, and (3) no adverse effect on the quality of the fMRI images. In the present study, we chose the marmoset as an example to verify the device.

The DPS consisted of two parts: the piezoelectric actuator unit generating the reciprocating force and the DPREU transforming and delivering the force to a target site. According to the inner size diameter of the scanner, the main body size of the executor of the DPS was set to 118 × 39 × 31 mm^3^. To ensure stable fixation of the DPS in the holder, the surface attached to the scanner holder was shaped into an arc matching the size of the holder. Additionally, the stimulation surface was angled at 30° to the horizontal plane, providing a comfortable position for the marmosets' forearm and hand.

We used three vibration frequencies (1, 5, and 10 Hz) to assess the output performance of the DPS. As shown in Figure 4b, the output forces were 30.52 (1 Hz), 31.49 (5 Hz), and 33.05 (10 Hz). The force increased by approximately 8% as the stimulation frequency increased from 1 to 10 Hz. The temporal characteristics of the DPS executor are illustrated in Figure 4c. The control system delays between the trigger signal from the controller and the onset of motion were 7.8 ms at 1 Hz and 7.5 ms at both 5 Hz and 10 Hz (blue line in Figure 4c). A certain amount of time was needed for the force to reach a stable output from the onset of motion, and this duration was defined as the motion rise time. The control system delay plus the motion rise time constituted the DPS delay, which represented the time required for the output to reach a stable condition. This delay was 12.9 ms at 1 Hz, 12.4 ms at 5 Hz, and 11.9 ms at 10 Hz.

### 3.2 Evaluation of the Noise Introduced by the Device

To determine the effect of the device on fMRI signals, we compared the tSNR under both the stimulator‐on and the stimulator‐off conditions. Figure 5c shows the tSNR maps of the phantom under both conditions. The mean tSNRs of the central ROI were 553.87 (1 Hz), 560.01 (10 Hz), and 560.08 (off). There was no significant difference in the tSNR among these three conditions (*F* (2, 798) = 0.71, *p* = 0.49). Figure 5d shows the averaged time courses within the ROI for the three conditions (1 Hz, 10 Hz, and no stimulus). The shaded areas represent the standard error of the mean (SEM).

### 3.3 fMRI Results

**FIGURE 7:**
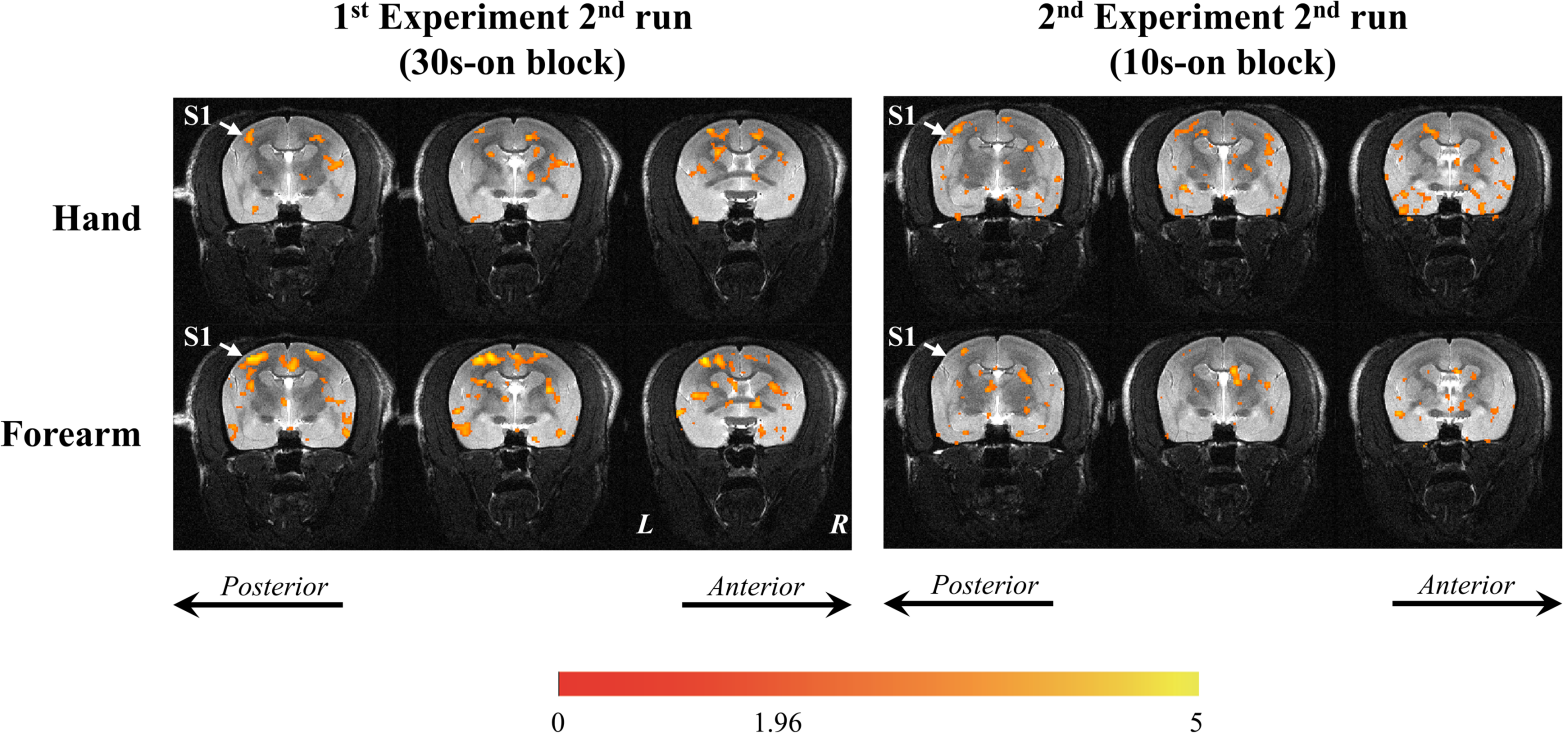
Hand and forearm somatosensory maps of marmoset #1. Activations are shown on the structural images of the marmoset. The increased CBV, which typically reflects increased neural activity, results in a decrease in MR signal intensity. Therefore, the colored areas in the images indicate regions with increased brain activity (negative response characters compared to baseline).

With the effect of the USPIO, the task‐related CBV‐weighted results were obtained. Based on the unique advantage of the precise stimulus locations provided by the DPS, the brain activity mapping of the hand and forearm was identified. With a threshold *t*‐value set to 1.96 (*p* < 0.05), both the three runs of the 30‐s‐on experiment and the four runs (run 1^st^, 2^nd^, 4^th^ and 5^th^) of the 10s‐on experiment exhibited significant activations in S1 in Marmoset #1. Additionally, Marmoset #2 and Marmoset #3 each showed significant S1 activation in two runs of the 10s‐on experiment. The activation maps of the hand and forearm of these 11 runs were extracted, and two representative results of Marmoset #1 were displayed on its structural images, as shown in the colored areas of Figure 7. The increased CBV, which typically reflects increased neural activity, results in a decrease in MR signal intensity. Therefore, the colored areas in the images indicate regions with increased brain activity (negative response characters compared to baseline).

**FIGURE 8:**
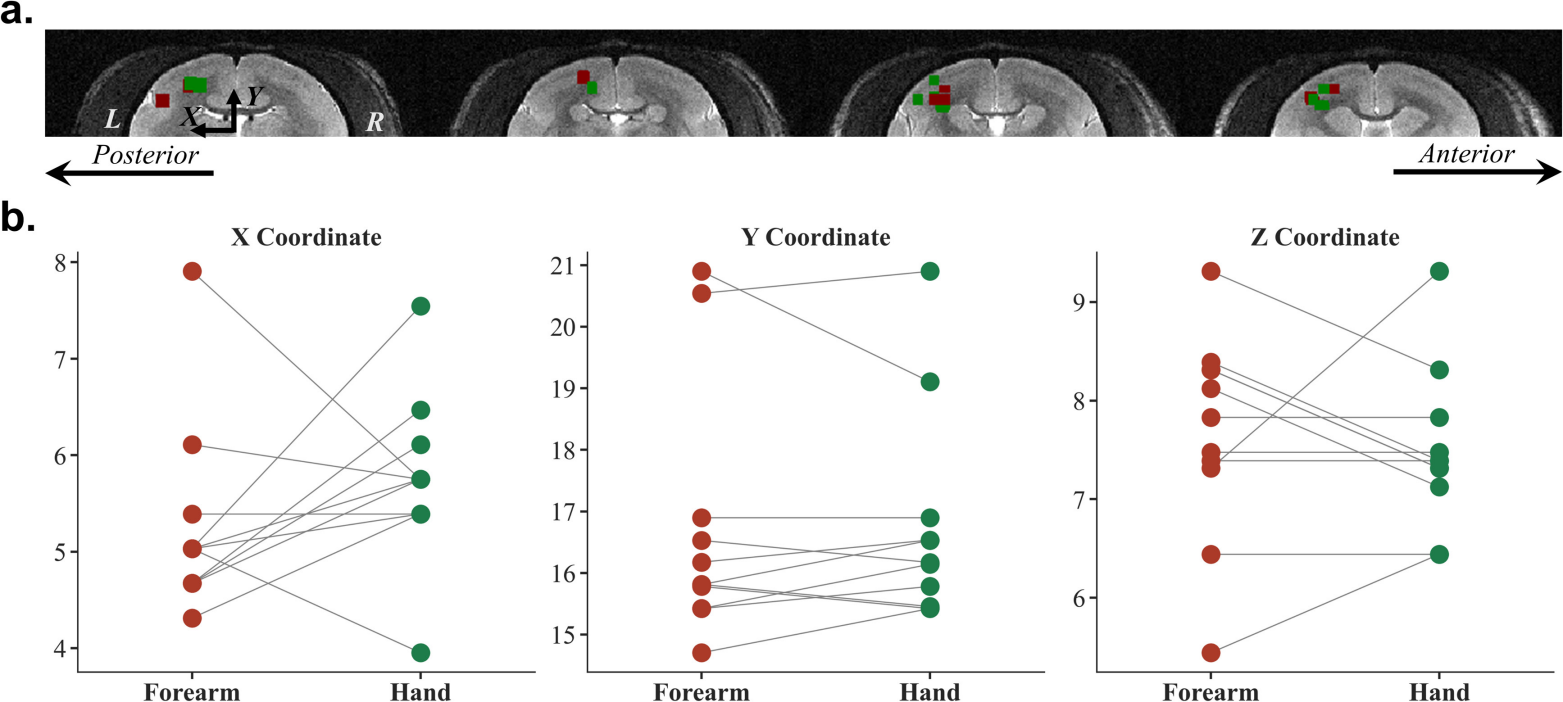
Center of mass of areas showing significant activity during hand and forearm stimulation in S1 (7 runs for Marmoset #1, 2 runs for Marmoset #2 and 2 runs for Marmoset #3). (a) Center of mass areas. The green dots represent the center of mass of the areas showing significant activity during hand stimulation in S1. The red dots represent the center of mass of the areas showing significant activity during forearm stimulation in S1. (b) Center of mass coordinates of the forearm and hand areas within S1. The circular dots represent the *x*, *y*, and *z* coordinates. The gray lines connect the hand and forearm dots from the same run. No significant differences were observed in the x, y and z coordinates.

The hand and forearm somatosensory locations were identified in the contralateral (left) lateral middle part of S1. In addition to the activation maps, the centers of mass maps of these 11 runs are shown in Figure 8a,b. The green dots represent the center of mass of the areas showing significant activity during hand stimulation in S1, whereas the red dots represent those for forearm stimulation. Figure 8a presents the center‐of‐mass locations in the 11 runs showing a high degree of overlap. A comparison of the coordinates of the center of mass of the hand and forearm in the 7 runs is shown in Figure 8b. The circular dots represent the xyz coordinates of the centers of mass for the hand and forearm representation areas. No significant differences were observed in the *x*, *y*, and *z* coordinates (*X* coordinate: *p* = 0.252, Y coordinate: *p* = 0.887, *Z* coordinate: *p* = 0.553).

**FIGURE 9:**
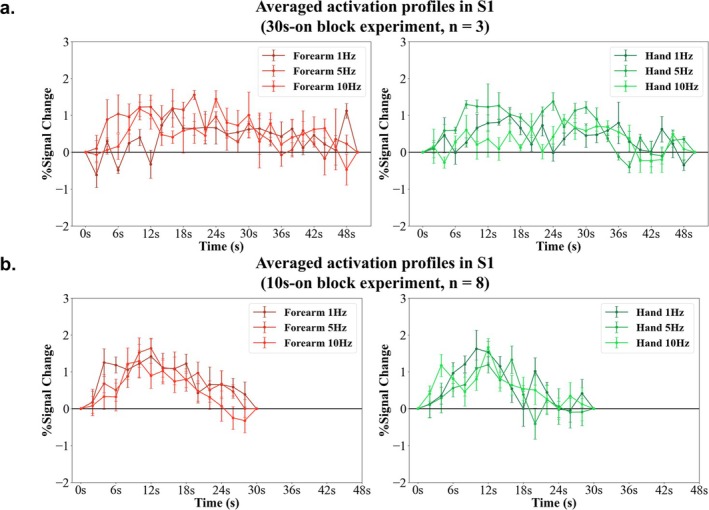
Activation profiles (reversed signal) at 10s‐on and 30s‐on block experiment during hand and forearm stimulation. The stimulation was provided at t = 0 point of each block. (a) Averaged activation profiles in S1 in 30s‐on block experiments across three runs of Marmoset #1. (b) Averaged activation profiles in S1 in 10s‐on block experiments across four runs of Marmoset #1, 2 runs of Marmoset #2 and 2 runs of Marmoset #3. The error bars indicate the standard error of the mean (SEM).

Figure 9a,b illustrates the activation profiles within S1 in response to stimulation frequencies for the hand and forearm, respectively. The three vibration frequencies delivered to the hand and forearm by the DPS system effectively elicited brain activity.

## 4 Discussion

In the present study, we developed an MR‐compatible tactile DPS for fMRI experiments aimed at investigating the somatosensory processing in small‐bodied animals. The DPS consisted of a dual‐point rocker–slider execution unit and a piezoelectric actuator unit and could generate steady skin indentation stimulation on the hand and forearm of marmosets in 7T fMRI environments. With the high spatial precision of the stimulation driven by the DPS, we investigated the somatosensory response of the forearm and hand to mechanical stimulation in the S1 of three anesthetized marmosets. The brain activity in response to forearm and hand stimulation confirmed that the stimuli were successfully applied to these regions. Overall, our findings provide new mechanical stimulation that shows high potential for investigating somatosensory processing in small‐bodied animals during UHF fMRI experiments.

The primary challenge in applying tactile stimulation to marmosets in a UHF fMRI scanner is the limited space. Both the small size of the scanner bore and the small size of the hand and forearm of marmosets restrict the size of the stimulator. While electrical stimulation has been used in several fMRI studies on marmosets [27, 28, 29], tactile signals are typically generated by mechanoreceptor capsules that respond to skin indentation, and electrical stimulation cannot effectively replicate this indentation [40]. Physical skin indentation is essential for reproducing tactile stimulation that occurs in the real world. To our knowledge, pneumatic stimulation has been the only method used to induce skin indentation to elicit somatosensory‐related brain activity in marmosets. However, due to the small size of the marmosets' body, high‐pressure air could affect areas beyond the intended target. To address this issue, we designed the DPS with two 2‐mm diameter probe channels to deliver tactile stimuli at specific locations, spaced 25 mm apart, to match the size of the marmosets' hand and forearm. With a unique double‐rocker mechanism, these two probes generated stimuli with an average force of 31.69 g at frequencies ranging from 1 to 10 Hz. The output motion demonstrated greater stability than pneumatic stimulation, ensuring stable and reproducible skin indentation. In the tSNR test, the device did not impair the quality of the MRI signals during EPI scans, enabling accurate detection of the brain activity in fMRI experiments. Furthermore, DPS had less and stable output delay than the pneumatic stimulation provided by the Galileo tactile simulator, whose rise time increased by 2 ms when the air tube length increased to 8′. The stable output of the DPS has great potential for investigating the temporal integration of tactile inputs using a two‐point delay paradigm. Due to the DPS's advantages in temporal and spatial precision, it is a promising new method for exploring somatosensory‐related brain activity in marmosets.

DPS functions in conjunction with UHF fMRI and provides a powerful tool for investigating somatosensory processing in marmosets. Previous studies using microelectrode recording techniques have demonstrated the topographic organization of S1 [22, 41]. To our knowledge, our study is the first to report brain activity in response to stimulation of marmosets' hand using a noninvasive imaging method. As shown in Figures 7 and 8, we identified the forearm and hand representation areas within S1. The topographic organization identified through electrophysiological recordings showed that the hand areas were positioned lateral and posterior to the forelimb area in S1 [41, 42]. In our study, the hand and forearm representation areas exhibited some overlap. The lack of a significant difference between the center‐of‐mass coordinates for the hand and forearm areas was likely caused by the proximity of the palm and forearm areas. The forearm area activated by our mechanical stimulation was smaller than that observed using a pneumatic device [30]. This difference was potentially caused by the more focused stimulation provided by our device. The ability of our device to target a more specific area was advantageous for exploring the somatosensory topographic map, especially considering the small size of the marmoset bodies. Additionally, the activity profiles in S1 among 3 frequencies also supported the ability of the DPS system in small‐bodied animal somatosensory research from temporal aspects. This localized and focused stimulation, combined with the high spatial resolution of UHF fMRI, provides a new approach for understanding the spatial distribution of the somatosensory cortex in marmosets.

Optimizing the timing of measurements such that CBV activity was estimated during the half‐life of USPIO could enhance both the sensitivity and spatial specificity of the results [43, 44]. The mean residence time of USPIO was 168 min in rats [45], and its half‐life was 118 min. Compared with our experiment, the contrast agent effect potentially decreased with time over the half‐life of USPIO in the final run of the 30s‐on experiment. Thus, since 10 s could significantly reduce the overall experimental time, we examined the 10s‐on blocks from the second day to determine if 10 s was sufficient to observe brain activation in response to tactile stimulation under anesthesia. Additionally, the shorter run time enabled the collection of more datasets in a single experiment; this aspect was especially important since the marmoset might wake up during the experiment even under anesthesia, and its movements could ruin the data from that run. The similarity between the center‐of‐mass maps from the 30s‐on and 10s‐on experiments indicated that the hand and forearm representation areas could be effectively determined using our developed device. Owing to its shorter on–off timing, the 10s‐on experiment provides a flexible and promising solution for observing marmoset somatosensory activity in future studies. These results also highlight the potential of using our device for precise and time‐efficient investigations into somatosensory processing.

The stimulations provided by the DPS system successfully evoked brain activity in marmosets, demonstrating its suitability for somatosensory research in small‐bodied animals. In addition to delivering variable frequencies, the DPS system can also generate different output forces by adjusting the number of piezoelectric bimorphs. Currently, each channel uses four bimorphs. In the future, we plan to develop an independent control system for the piezo bimorphs, adding force‐adjustable functionality to the DPS system. This would allow us to vary the number of active bimorphs per channel (from one to four), enabling investigations of brain responses to different force levels in small‐bodied animal somatosensory research.

## 5 Limitations

First, all images were acquired by a single MR scanner. Second, the current device used in the present study was developed in‐house, limiting its availability compared with commercially available devices. Finally, the experiment involved only three marmosets, which may not provide sufficient data to fully define a common topological relationship between the brain activity and the forearm and hand.

## 6 Conclusion

In conclusion, the DPS successfully elicited forearm and hand somatosensory‐related brain activity in anesthetized marmosets, as demonstrated by the fMRI experiments. This device showed its advantage in studying somatosensory‐related brain activity using UHF fMRI.

## Data Availability

The data that support the findings of this study are available from the corresponding author upon reasonable request.

## References

[1] Y. Yu , L. Huber , J. Yang , et al., “Layer‐Specific Activation of Sensory Input and Predictive Feedback in the Human Primary Somatosensory Cortex,” Science Advances 5 (2019): eaav9053. 31106273 10.1126/sciadv.aav9053 PMC6520017

[2] J. Yang , P. J. Molfese , Y. Yu , et al., “Different Activation Signatures in the Primary Sensorimotor and Higher‐Level Regions for Haptic Three‐Dimensional Curved Surface Exploration,” NeuroImage 231 (2021): 231, 10.1016/j.neuroimage.2021.117754. PMC11822888 33454415

[3] Y. Yu , L. Huber , J. Yang , et al., “Layer‐Specific Activation in Human Primary Somatosensory Cortex During Tactile Temporal Prediction Error Processing,” NeuroImage 248 (2022): 118867, 10.1016/j.neuroimage.2021.118867. 34974114 PMC11835052

[4] M. Khateb , J. Schiller , and Y. Schiller , “Feedforward Motor Information Enhances Somatosensory Responses and Sharpens Angular Tuning of Rat S1 Barrel Cortex Neurons,” Elife 6 (2017): e21843, 10.7554/eLife.21843.001. 28059699 PMC5271607

[5] E. Zagha , A. E. Casale , R. N. S. Sachdev , M. J. McGinley , and D. A. McCormick , “Motor Cortex Feedback Influences Sensory Processing by Modulating Network State,” Neuron 79, no. 3 (2013): 567–578, 10.1016/j.neuron.2013.06.008. 23850595 PMC3742632

[6] J. P. Capitanio and M. E. Emborg , “Contributions of Non‐Human Primates to Neuroscience Research,” Lancet 371, no. 9618 (2008): 1126–1135, 10.1016/S0140-6736(08)60489-4. 18374844

[7] D. H. O'Connor , L. Krubitzer , and S. Bensmaia , “Of Mice and Monkeys: Somatosensory Processing in Two Prominent Animal Models,” Progress in Neurobiology 201 (2021): 201, 10.1016/j.pneurobio.2021.102008. PMC8096687 33587956

[8] J. Walker , J. MacLean , and N. G. Hatsopoulos , “The Marmoset as a Model System for Studying Voluntary Motor Control,” Developmental Neurobiology 77, no. 3 (2017): 273–285, 10.1002/dneu.22461. 27739220

[9] T. Umeda , M. Koizumi , Y. Katakai , R. Saito , and K. Seki , “Decoding of Muscle Activity From the Sensorimotor Cortex in Freely Behaving Monkeys,” NeuroImage 197 (2019): 512–526, 10.1016/j.neuroimage.2019.04.045. 31015029

[10] A. C. Silva , “Anatomical and Functional Neuroimaging in Awake, Behaving Marmosets,” Developmental Neurobiology 77, no. 3 (2017): 373–389, 10.1002/dneu.22456. 27706916 PMC5318267

[11] J. M. Burkart and C. Finkenwirth , “Marmosets as Model Species in Neuroscience and Evolutionary Anthropology,” Neuroscience Research 93 (2015): 8–19, 10.1016/j.neures.2014.09.003. 25242577

[12] S. G. Solomon and M. G. P. Rosa , “A Simpler Primate Brain: The Visual System of the Marmoset Monkey,” Front Neural Circuits 8 (2014): 8(AUG), 10.3389/fncir.2014.00096. 25152716 PMC4126041

[13] J. H. Kaas , “Comparative Functional Anatomy of Marmoset Brains,” ILAR Journal 61, no. 2‐3 (2020): 260–273, 10.1093/ilar/ilaa026. 33550381 PMC9214571

[14] L. Huber , D. H. Y. Tse , C. J. Wiggins , et al., “Ultra‐High Resolution Blood Volume fMRI and BOLD fMRI in Humans at 9.4 T: Capabilities and Challenges,” NeuroImage 178 (2018): 769–779, 10.1016/j.neuroimage.2018.06.025. 29890330 PMC6100753

[15] L. Huber , B. A. Poser , A. L. Kaas , et al., “Validating Layer‐Specific VASO Across Species,” NeuroImage 237 (2021): 118195, 10.1016/j.neuroimage.2021.118195. 34038769

[16] J. Yang , L. Huber , Y. Yu , and P. A. Bandettini , “Linking Cortical Circuit Models to Human Cognition With Laminar fMRI,” Neuroscience & Biobehavioral Reviews 128 (2021): 467–478, 10.1016/j.neubiorev.2021.07.005. 34245758 PMC12906289

[17] M. Guidi , L. Huber , L. Lampe , C. J. Gauthier , and H. E. Möller , “Lamina‐Dependent Calibrated BOLD Response in Human Primary Motor Cortex,” NeuroImage 141 (2016): 250–261, 10.1016/j.neuroimage.2016.06.030. 27364473

[18] J. H. Kaas , “What, if Anything, Is SI? Organization of First Somatosensory Area of Cortex,” Physiological Reviews 63, no. 1 (1983): 206–231, 10.1152/physrev.1983.63.1.206. 6401864

[19] J. L. Reed , H. X. Qi , Z. Zhou , et al., “Response Properties of Neurons in Primary Somatosensory Cortex of Owl Monkeys Reflect Widespread Spatiotemporal Integration,” Journal of Neurophysiology 103, no. 4 (2010): 2139–2157, 10.1152/jn.00709.2009. 20164400 PMC2853283

[20] C. Bowes , M. Burish , C. Cerkevich , and J. Kaas , “Patterns of Cortical Reorganization in the Adult Marmoset After a Cervical Spinal Cord Injury,” Journal of Comparative Neurology 521, no. 15 (2013): 3451–3463, 10.1002/cne.23360. 23681952 PMC3977742

[21] H. X. Qi , D. C. Lyon , and J. H. Kaas , “Cortical and Thalamic Connections of the Parietal Ventral Somatosensory Area in Marmoset Monkeys (*Callithrix jacchus*),” Journal of Comparative Neurology 443, no. 2 (2002): 168–182, 10.1002/cne.10113. 11793354

[22] K. J. Huffman and L. Krubitzer , “Area 3a: Topographic Organization and Cortical Connections in Marmoset Monkeys,” Cerebral Cortex 11, no. 9 (2001): 849–867, 10.1093/cercor/11.9.849. 11532890

[23] M. Carlson , M. F. Huerta , C. G. Cusick , and J. H. Kaas , “Studies on the Evolution of Multiple Somatosensory Representations in Primates: The Organization of Anterior Parietal Cortex in the New World Callitrichid, Saguinus,” Journal of Comparative Neurology 246, no. 3 (1986): 409–426, 10.1002/cne.902460309. 3084599

[24] J. L. Reed , H. X. Qi , and J. H. Kaas , “Spatiotemporal Properties of Neuron Response Suppression in Owl Monkey Primary Somatosensory Cortex When Stimuli Are Presented to Both Hands,” Journal of Neuroscience 31, no. 10 (2011): 3589–3601, 10.1523/JNEUROSCI.4310-10.2011. 21389215 PMC3063385

[25] J. W. Lane , P. J. Fitzgerald , J. M. Yau , I. Pembeci , and S. S. Hsiao , “A Tactile Stimulator for Studying Passive Shape Perception,” Journal of Neuroscience Methods 185, no. 2 (2010): 221–229, 10.1016/j.jneumeth.2009.09.025. 19800916 PMC2815267

[26] S. S. Kim , M. Gomez‐Ramirez , P. H. Thakur , and S. S. Hsiao , “Multimodal Interactions Between Proprioceptive and Cutaneous Signals in Primary Somatosensory Cortex,” Neuron 86, no. 2 (2015): 555–566, 10.1016/j.neuron.2015.03.020. 25864632 PMC4409561

[27] D. Papoti , C. C. C. Yen , J. B. Mackel , H. Merkle , and A. C. Silva , “An Embedded Four‐Channel Receive‐Only RF Coil Array for fMRI Experiments of the Somatosensory Pathway in Conscious Awake Marmosets,” NMR in Biomedicine 26, no. 11 (2013): 1395–1402, 10.1002/nbm.2965. 23696219 PMC4200535

[28] J. V. Liu , Y. Hirano , G. C. Nascimento , B. Stefanovic , D. A. Leopold , and A. C. Silva , “FMRI in the Awake Marmoset: Somatosensory‐Evoked Responses, Functional Connectivity, and Comparison With Propofol Anesthesia,” NeuroImage 78 (2013): 186–195, 10.1016/j.neuroimage.2013.03.038. 23571417 PMC3778909

[29] Y. Hirano , C. C. Yen , J. V. Liu , et al., “Investigation of the BOLD and CBV fMRI Responses to Somatosensory Stimulation in Awake Marmosets (*Callithrix jacchus*),” NMR in Biomedicine 31, no. 3 (2018): e3864, 10.1002/nbm.3864. PMC5841465 29285809

[30] J. C. Clery , Y. Hori , D. J. Schaeffer , J. S. Gati , J. Andrew Pruszynski , and S. Everling , “Whole Brain Mapping of Somatosensory Responses in Awake Marmosets Investigated With Ultra‐High‐Field fMRI,” Journal of Neurophysiology 124, no. 6 (2020): 1900–1913, 10.1152/jn.00480.2020. 33112698

[31] V. Yem and H. Kajimoto , “Comparative Evaluation of Tactile Sensation by Electrical and Mechanical Stimulation,” IEEE Transactions on Haptics 10, no. 1 (2017): 130–134, 10.1109/TOH.2016.2605084. 28113382

[32] E. R. Gizewski , O. Koeze , K. Uffmann , A. De Greiff , M. E. Ladd , and M. Forsting , “Cerebral Activation Using a MR‐Compatible Piezoelectric Actuator With Adjustable Vibration Frequencies and In Vivo Wave Propagation Control,” NeuroImage 24, no. 3 (2005): 723–730, 10.1016/j.neuroimage.2004.09.015. 15652307

[33] S. T. Francis , E. F. Kelly , R. Bowtell , W. J. R. Dunseath , S. E. Folger , and F. McGlone , “fMRI of the Responses to Vibratory Stimulation of Digit Tips,” NeuroImage 11, no. 3 (2000): 188–202, 10.1006/nimg.2000.0541. 10694461

[34] G. S. Harrington and J. Hunter Downs , “FMRI Mapping of the Somatosensory Cortex With Vibratory Stimuli: Is There a Dependency on Stimulus Frequency?,” Brain Research 897, no. 1‐2 (2001): 188–192, 10.1016/S0006-8993(01)02139-4. 11282375

[35] M. A. Schweisfurth , J. Frahm , and R. Schweizer , “Individual fMRI Maps of All Phalanges and Digit Bases of All Fingers in Human Primary Somatosensory Cortex,” Frontiers in Human Neuroscience 8 (2014): 658, 10.3389/fnhum.2014.00658. 25228867 PMC4151507

[36] J. Wu , C. Wang , L. Wang , et al., “Development of a Piezoelectric Actuated Tactile Stimulation Device for Population Receptive Field Mapping in Human Somatosensory Cortex With fMRI,” Journal of Magnetic Resonance Imaging 56, no. 4 (2022): 1055–1065, 10.1002/jmri.28173. 35324031

[37] R. W. Cox , “AFNI: Software for Analysis and Visualization of Functional Magnetic Resonance Neuroimages,” Computers and Biomedical Research 29, no. 3 (1996): 162–173, 10.1006/cbmr.1996.0014. 8812068

[38] F. P. Leite and J. B. Mandeville , “Characterization of Event‐Related Designs Using BOLD and IRON fMRI,” NeuroImage 29, no. 3 (2006): 901–909, 10.1016/j.neuroimage.2005.08.022. 16213164

[39] F. P. Leite , D. Tsao , W. Vanduffel , et al., “Repeated fMRI Using Iron Oxide Contrast Agent in Awake, Behaving Macaques at 3 Tesla,” NeuroImage 16, no. 2 (2002): 283–294, 10.1006/nimg.2002.1110. 12030817

[40] A. Handler and D. D. Ginty , “The Mechanosensory Neurons of Touch and Their Mechanisms of Activation,” Nature Reviews Neuroscience 22, no. 9 (2021): 521–537, 10.1038/s41583-021-00489-x. 34312536 PMC8485761

[41] L. A. Krubitzer and J. H. Kaas , “Responsiveness and Somatotopic Organization of Anterior Parietal Field 3b and Adjoining Cortex in Newborn and Infant Monkeys,” Somatosensory & Motor Research 6, no. 2 (1988): 179–205, 10.3109/08990228809144673. 3242345

[42] P. E. Garraghty , T. P. Pons , and J. H. Kaas , “Ablations of Areas 3b (SI Proper) and 3a of Somatosensory Cortex in Marmosets Deactivate the Second and Parietal Ventral Somatosensory Areas,” Somatosensory & Motor Research 7, no. 2 (1990): 125–135, 10.3109/08990229009144703. 2116056

[43] C. Corot , P. Robert , J. M. Idée , and M. Port , “Recent Advances in Iron Oxide Nanocrystal Technology for Medical Imaging,” Advanced Drug Delivery Reviews 58, no. 14 (2006): 1471–1504, 10.1016/j.addr.2006.09.013. 17116343

[44] S. G. Kim , N. Harel , T. Jin , T. Kim , P. Lee , and F. Zhao , “Cerebral Blood Volume MRI With Intravascular Superparamagnetic Iron Oxide Nanoparticles,” NMR in Biomedicine 26, no. 8 (2013): 949–962, 10.1002/nbm.2885. 23208650 PMC3700592

[45] C. Chambon , O. Clement , A. Le Blanche , E. Schouman‐Claeys , and G. Frija , “Superparamagnetic Iron Oxides as Positive MR Contrast Agents: In Vitro and In Vivo Evidence,” Magnetic Resonance Imaging 11, no. 4 (1993): 509–519, 10.1016/0730-725X(93)90470-X. 8316064

